# Temporal Onset Focal Seizures Induced by Intermittent Photic Stimulation

**DOI:** 10.3389/fneur.2021.715236

**Published:** 2021-08-30

**Authors:** Yue Niu, Pan Gong, Xianru Jiao, Haipo Yang, Zhixian Yang

**Affiliations:** Department of Pediatrics, Peking University First Hospital, Beijing, China

**Keywords:** photosensitive epilepsy, focal seizures, temporal lobe epilepsy, photoparoxysmal response, photo-convulsive response

## Abstract

The study aimed to review the clinical, radiological, and pathological findings and electroencephalogram (EEG) of pediatric epilepsy patients with temporal onset focal seizures induced by intermittent photic stimulation (IPS). Four patients with temporal onset photosensitivity focal seizures were analyzed. Three (75%) of the four patients were female. The average age of seizure-onset was 4.4 years. The interictal EEG showed both generalized and focal spike and waves in one patient and focal or multifocal spike and waves alone in three patients. Photoparoxysmal response (PPR) was evoked in all patients and showed generalized discharges (patients 2–4), both generalized and posterior discharges (patient 1). Both generalized and focal discharges could coexist in interictal discharges and PPR. The sensitive frequencies of PPR and photoconvulsive response (PCR) were 12–30 and 10–16 Hz, respectively, which were close to the occipital rhythm. In all patients, the onset of PCR was recorded, namely, the left anterior and mesial temporal lobe (TL), the left posterior TL, and the whole left TL, which showed two forms: the seizure of two patients was the onset of slow waves in the temporal area without spreading generalized discharges (patients 1 and 4), and the other one was fast rhythmic activities in the temporal area, spreading to the occipital area or gradually evolving into the generalized discharges (patients 2 and 3). During follow-up, except for patient 3 who had occasional seizures, the seizures of the remaining patients were under control. Temporal onset focal seizures could be induced by IPS. Temporal onset photosensitivity seizures were mostly easy to control with antiseizure drugs.

## Introduction

Seizures triggered by photic stimulation are usually associated with a subtype of genetic (idiopathic) generalized epilepsy, such as, childhood absence epilepsy, juvenile absence epilepsy, and juvenile myoclonic epilepsy (1). In the electroencephalogram (EEG), the photoparoxysmal response (PPR) pattern induced by intermittent photic stimulation (IPS) is usually manifested as generalized polyspikes and slow waves. There are also some reports of focal seizures induced by IPS, most of which are caused by the high excitability of the occipital cortex (2, 3). In 1996, it was reported that a patient diagnosed with temporal lobe epilepsy was detected as having a photosensitive temporal seizure (4). In 2021, a study reported a case of temporal lobe focal seizure induced by IPS, but no confirmed temporal lobe epilepsy (5). However, detailed EEG and clinical information

on photosensitive focal temporal seizures is scarce (5, 6). Therefore, we reviewed 243 patients whose photoconvulsive response (PCR) were recorded by video-EEG from 2010 to 2019 at our center and found that four patients were recorded photosensitive focal seizures originating from the temporal lobe.

## Patients and Methods

### Ethics Statement

This study was approved by the Biomedical Research Ethical Committee of Peking University First Hospital. The individuals or their parents in this manuscript have given their written informed consent to publish the case details.

### Methods

We searched the EEG database using the keyword “PCR” and found 243 PCR EEG recordings of 67,275 video-EEG recordings in the Pediatric EEG Department of Peking University First Hospital from 2010 to 2019. Finally, we identified four patients of temporal onset focal seizures induced by IPS. We re-evaluated the EEG of four patients and graded them according to the standardization of the IPS procedure. We reviewed clinical data, including gender, age at seizure onset, seizure types, perinatal and personal history, family history, treatment, and other relevant clinical data. Brain magnetic resonance imaging (MRI) and neuropsychological assessment were also reviewed. Patients were followed up at the pediatric neurology clinic or *via* telephone.

A 32-channel digital video-EEG system (Nihon Kohden; Japan) was used to complete 4-h monitoring (covering sleep and wakefulness) and were placed scalp electrodes in accordance with the international standard lead 10–20 system. According to international standards and actual situation, IPS was performed in a dimly lit environment using a round lamp with a diameter of 10 cm. Eye states were divided into three categories, namely, eyes opened, eyes closed, and eyes closure. Each group of eye states was followed by an increase in the stimulation frequency from 2 to 20 Hz, and then a decrease from 60 to 20 Hz, increasing by 2 Hz or decreasing by 10 Hz, respectively. Five-second trains of flashes for each frequency were delivered, at intervals of 10 s. When IPS induced seizures, the IPS test should be stopped immediately. PPR was graded on a scale of 1 to 3, from temporoparietooccipital epileptiform discharges (grade I), starting temporoparietooccipital and spreading to frontal regions (grade II), and to generalized epileptiform discharges (grade III) (7). In both video-EEG monitoring and our research, all EEGs were evaluated by at least two experienced neurophysiologists.

## Results

### Clinical Features

Clinical features of affected four individuals are summarized in Table 1. Three patients were female. All four patients were born after a normal pregnancy and uneventful delivery. Patient 1 had a history of three febrile seizures. The mother patient 2 had a history of febrile seizures. The average age of seizure onset was 4.4 years, ranging from 1 year and 7 months to 6 years. The seizure types were roughly classified according to medical records, including generalized tonic-clonic seizures (GTCS, 2/4) and focal to bilateral tonic-clonic seizures (FBTCS, 2/4). Patient 1 had more frequent clinical seizures, occurring every 2 weeks. Patient 2 had three identical seizures in total. At first, the parents of patient 3 did not think that the child had convulsions. They regarded the seizures of patient 3 as a cardiovascular disease and were hospitalized in the pediatric cardiovascular department of our hospital. The seizure of patient 3 was described as a brief loss of consciousness by his parents. During this time, he completed an EEG examination and was recorded IPS-induced seizures. Furthermore, upon inquiry of the medical history of this patient, it was traced back to the abovementioned seizures twice within 2 years. The frequency of seizures in patient 4 was one to two seizures per year.

**Table 1:** Summary of the clinical features of four individuals.

	**Gender**	**Age of onset**	**Age at EEG monitoring**	**ASMs used before monitoring**	**ASMs at EEG monitoring**	**History of FS**	**Family history of FS**	**Brain MRI**	**Cognitive and behavioral development**
Patient 1	F	5 years	6 years	CBZ, VPA, LTG	CBZ, VPA	+	–	Normal	Normal
Patient 2	F	5 years	11 years	–	LTG	–	+	Normal	Normal
Patient 3	M	6 years	8 years	–	VPA	–	–	Normal	Normal
Patient 4	F	1 year 7 months	9 years	LEV, OXC	OXC, VPA	–	–	Normal	Normal

*F, female; M, male; EEG, electroencephalogram; ASMs, antiseizure medications; CBZ, carbamazepine; VPA, valproic acid; LTG, lamotrigine; LEV, levetiracetam; OXC, oxcarbazepine; CZP, clonazepam; TPM, topiramate; FS, febrile seizures; MRI, magnetic resonance imaging; N, normal*.

### EEG Findings and Brain MRI

**Figure 1:**
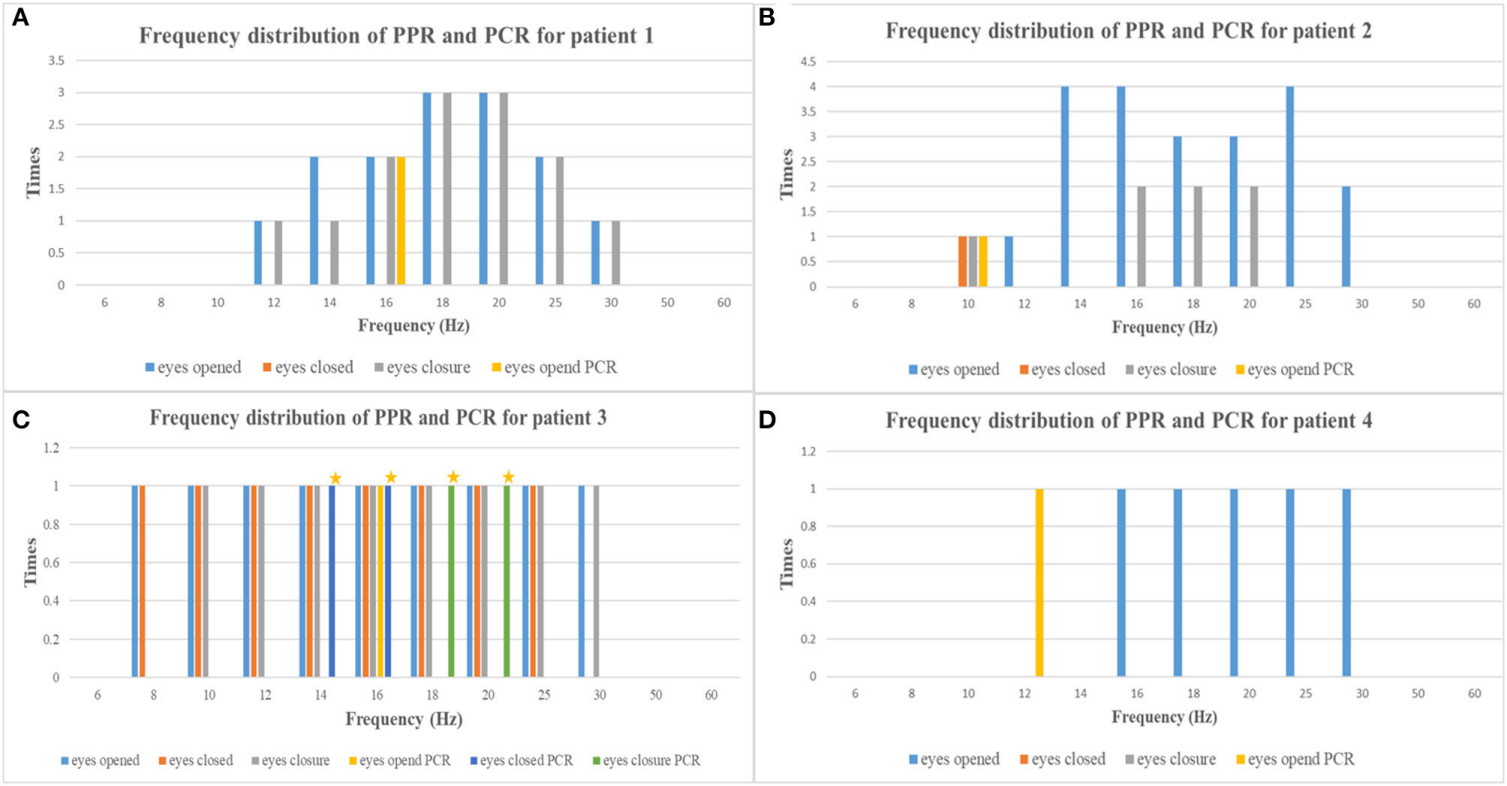
The frequency distribution of PPR and PCR of each patient [**(A)** patient 1, **(B)** patient 2, **(C)** patient 3, **(D)** patient 4; yellow asterisks, myoclonic seizures] (PPR, photoparoxysmal response; PCR, photoconvulsive response).

The average age at the time of EEG monitoring was 8.5 years with a range of 6–11 years. EEG findings of individuals are summarized in Table 2, and the frequency distribution of PPR and PCR for each patient is shown in Figure 1. A normal background activity was observed in all patients.

**Table 2:** Summary of EEG findings of individuals with temporal onset focal seizure induced by IPS.

**No**.	**EEG background**	**Interictal EDs**	**PPR grade**	**Frequency distribution of PPR (Hz)**	**Seizure type of PCR**	**Electrographic onset**	**Frequency distribution of PCR (Hz)**	**Ictal symptom**
Patient 1	Normal	GSW, occasionally in the left frontotemporal	III III I, III I	12–25 14–20 18–30 12–25	FS FS	Left anterior mesial temporal	16 16	Aura of vision hallucinations, decreased consciousness
Patient 2	Normal	Right mesial and posterior temporal, occasionally in the left temporal	III III III III	14–30 14–25 14–20 12–25	FBTCS	Left posterior temporal	10	Consciously eyes staring and then quickly followed by generalized limbs convulsions with loss of consciousness
Patient 3	Normal	Rolandic area	III	8–30	FS, myoclonus seizures	Left temporal	16 14–20	Decreased consciousness, forced turning of the head, and eyes deviation to the right side
Patient 4	Normal	Right occipital, right anterior mesial temporal	III	16–30	FS	Left temporal	12	Decreased consciousness, eyes squinted to 1 side with smacking swallowing action

*EEG, electroencephalographic; IPS, intermittent photic stimulation; EDs, epileptiform discharges; GSW, generalized spike and waves; PPR, photoparoxysmal response; PCR, photoconvulsion response; FS, focal seizures; FBTCS, focal to bilateral tonic-clonic seizures; I, temporoparietooccipital epileptiform discharges; II, temporoparietooccipital and spreading to frontal regions; III, generalized epileptiform discharges*.

The interictal EEG showed both generalized and focal left frontotemporal spike and waves in only patient 1, focal or multifocal spike and waves alone in other three patients. In the three patients with interictal focal or multifocal discharges, the locations included unilateral or bilateral mesial and posterior temporal region in patient 2, rolandic area alone in patient 3, and right occipital and anterior mesial temporal region in patient 4.

**Figure 2:**
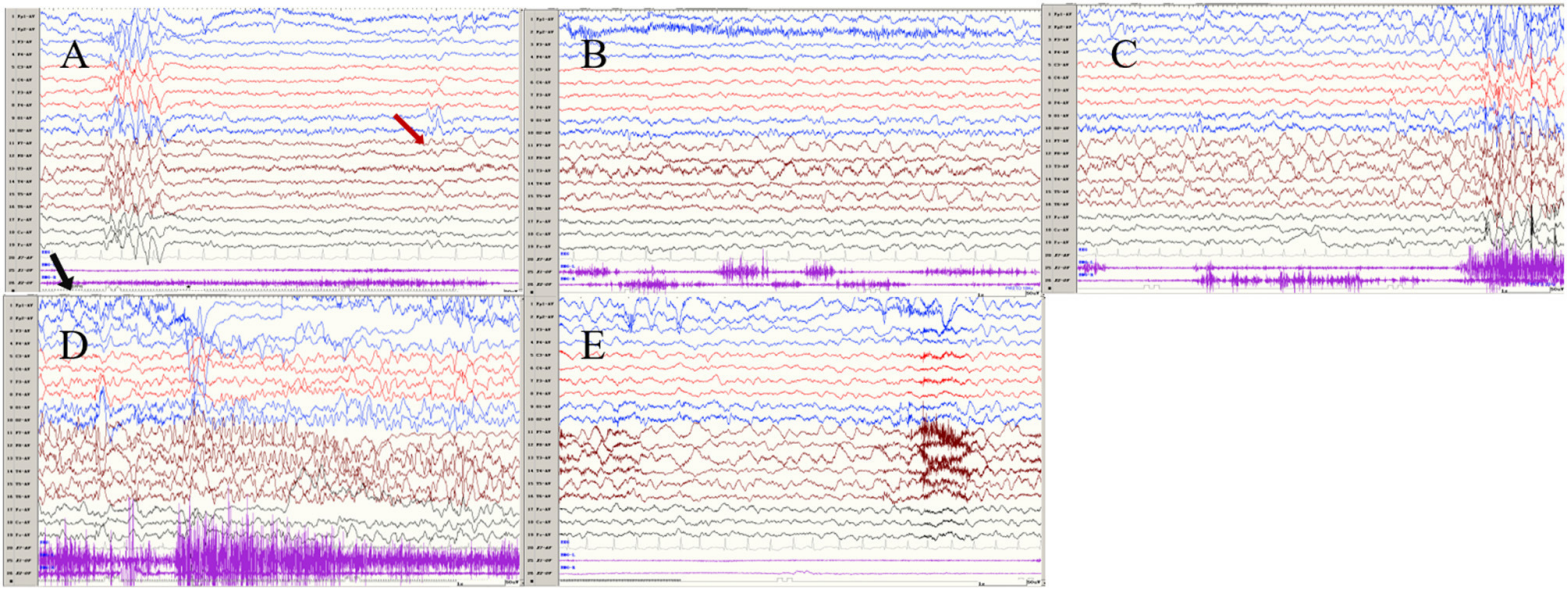
The ictal EEG of left temporal onset focal seizure induced by IPS in patient 1 **(A**–**E)** the initial 1.5–2-Hz slow waves in the left anterior and mesial temporal area → the spikes mixed waves, and then medium amplitude delta waves in the left temporal area. (The red arrow represents the origin of the seizure; the black arrow represents the starting point of the IPS.) (EEG, electrocardiogram; IPS, intermittent photic stimulation; X1, left deltoid; X2, right deltoid).

PPR was evoked by eyes opened IPS in all patients, by eyes closed IPS in two patients (patients 2 and 3), and by eyes closure IPS in three patients (patients 1, 2, and 3), respectively. The sensitive frequency of PPR was mainly concentrated in the range of 12–30 Hz. At the last follow-up, patient 1 underwent a total of five times of video-EEG examinations every 6 months or 1 year and four times of video-EEG–induced PPR (Figure 2). Among the four-time PPRs, the first two were mainly generalized discharges (grade III), the third time had both posterior and generalized discharges, and the fourth time only showed posterior discharges dominated by occipital discharges (grade I). The EEG of patient 2 was also induced PPR for four times, all of which were predominantly generalized discharges (grade III), while the EEGs of patients 3 and 4 were induced only one-time PPR, both of which were generalized discharges (grade III).

Ictal symptoms varied (Table 2). All patients were accompanied by decreased consciousness or complete loss of consciousness. Among them, patient 1 presented with “aura of vision hallucination” and decreased consciousness, patient 2 showed conscious staring at the eyes, followed by generalized limb convulsions with loss of consciousness, patient 3's ictal symptom manifested as decreased consciousness, forced turning of the head, and eye deviation to the right side, and patient 4 presented with decreased consciousness, eyes squinted to one side with smacking swallowing action. Interestingly, patient 1 was found for two-time focal seizures induced by IPS, both of which were evoked by eyes opened IPS with 16 Hz (Figure 2). The ictal EEG of patient 2 was induced at 16 Hz with eyes opened, while the focal seizures of patients 3 and 4 were induced at 16 and 12 Hz with eyes opened, respectively. In patient 3, myoclonic seizures were also monitored and the corresponding ictal EEG showed generalized spike and waves with a frequency of 14–20 Hz.

**Figure 3:**
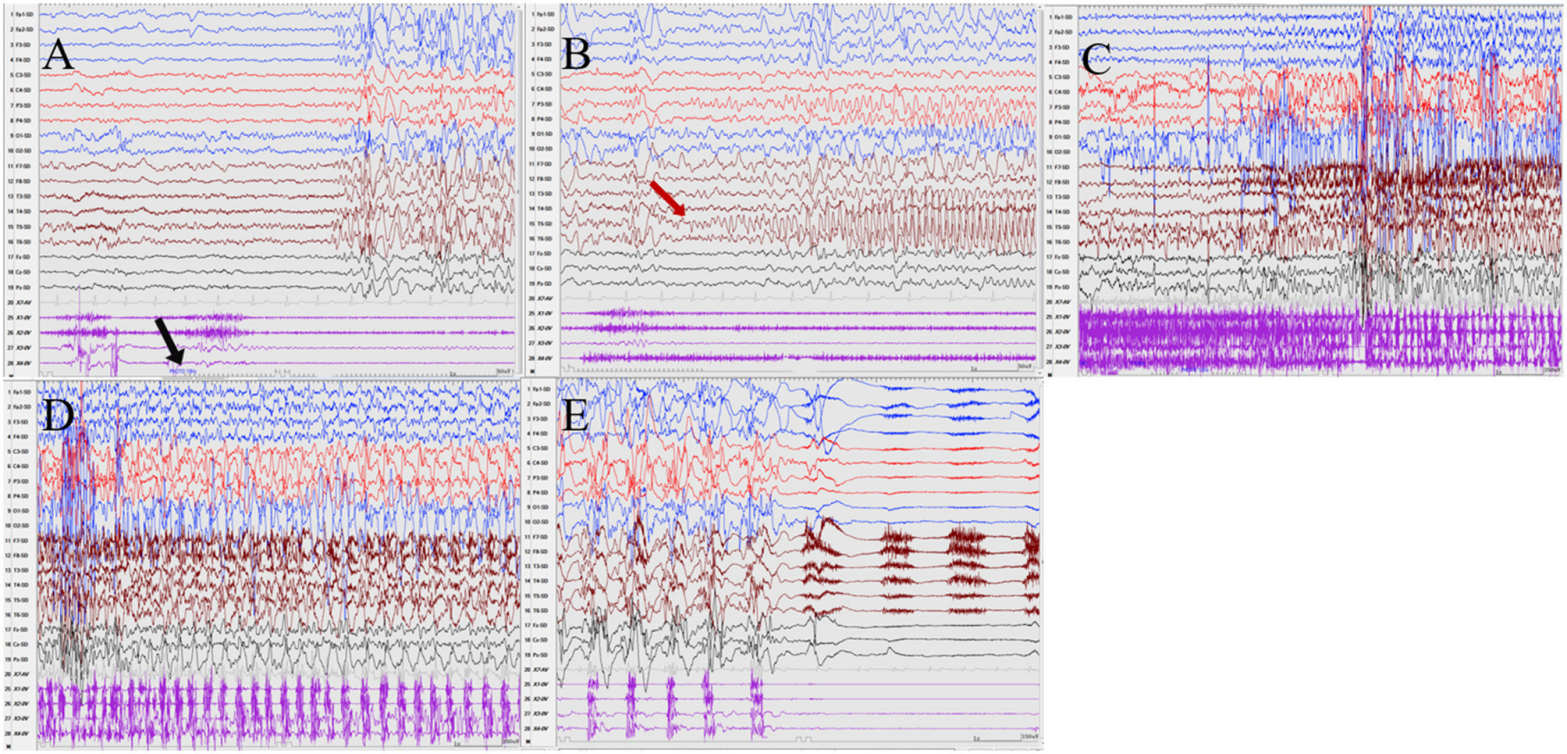
The ictal EEG of patient 2 **(A**–**E)** 3–3.5 Hz generalized spike and waves → left posterior temporal spikes rhythm (T5) → spread to the occipital and left parietal regions (O1, P3) → generalized fast wave rhythm → slow wave insertion. (The red arrow represents the origin of the seizure; the black arrow represents the starting point of the IPS.) (EEG, electrocardiogram; IPS, intermittent photic stimulation; X1, left deltoid; X2, right deltoid; X3, left quadriceps; X4, right quadriceps).

**Figure 4:**
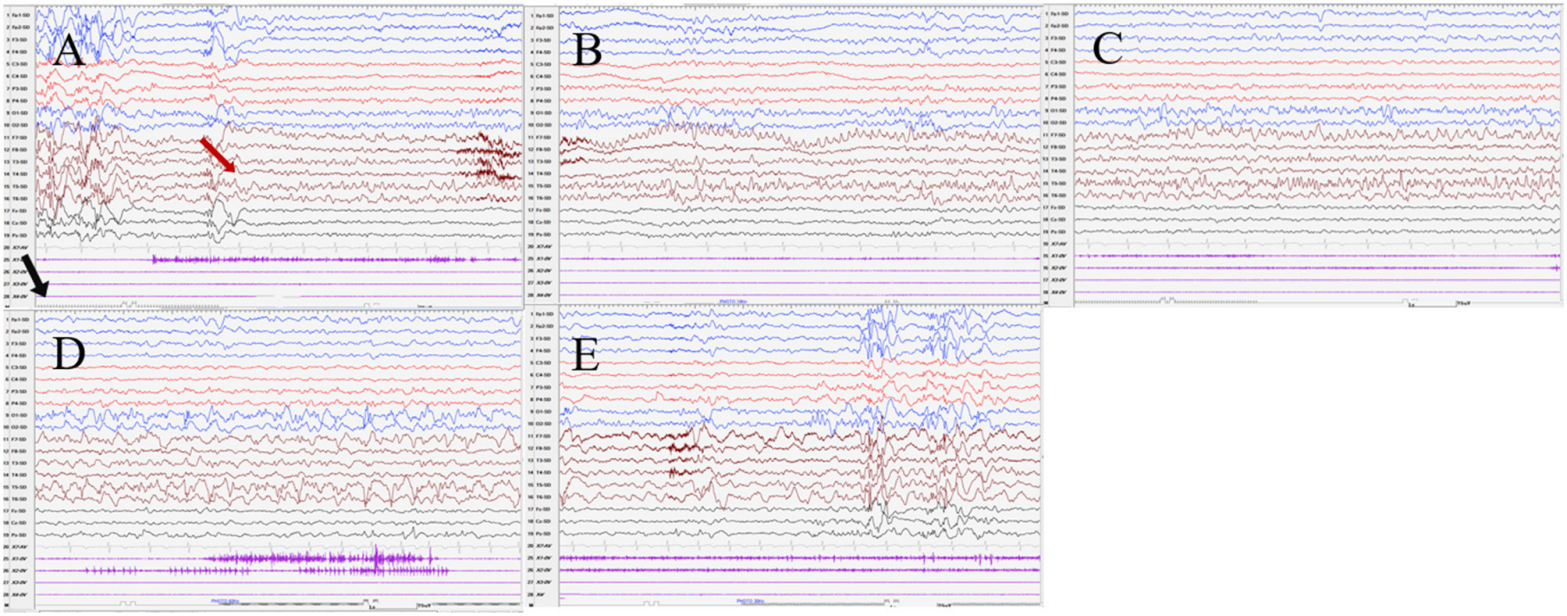
The ictal EEG of patient 3 **(A**–**E)** 3–3.5 Hz generalized spike and waves → 7–8 Hz mixed rhythm in the left temporal region spreading to the left occipital region → slowing down to delta waves of the frequency. (The red arrow represents the origin of the seizure; the black arrow represents the starting point of the IPS.) (EEG, electrocardiogram; IPS, intermittent photic stimulation; X1, left deltoid; X2, right deltoid; X3, left quadriceps; X4, right quadriceps).

**Figure 5:**
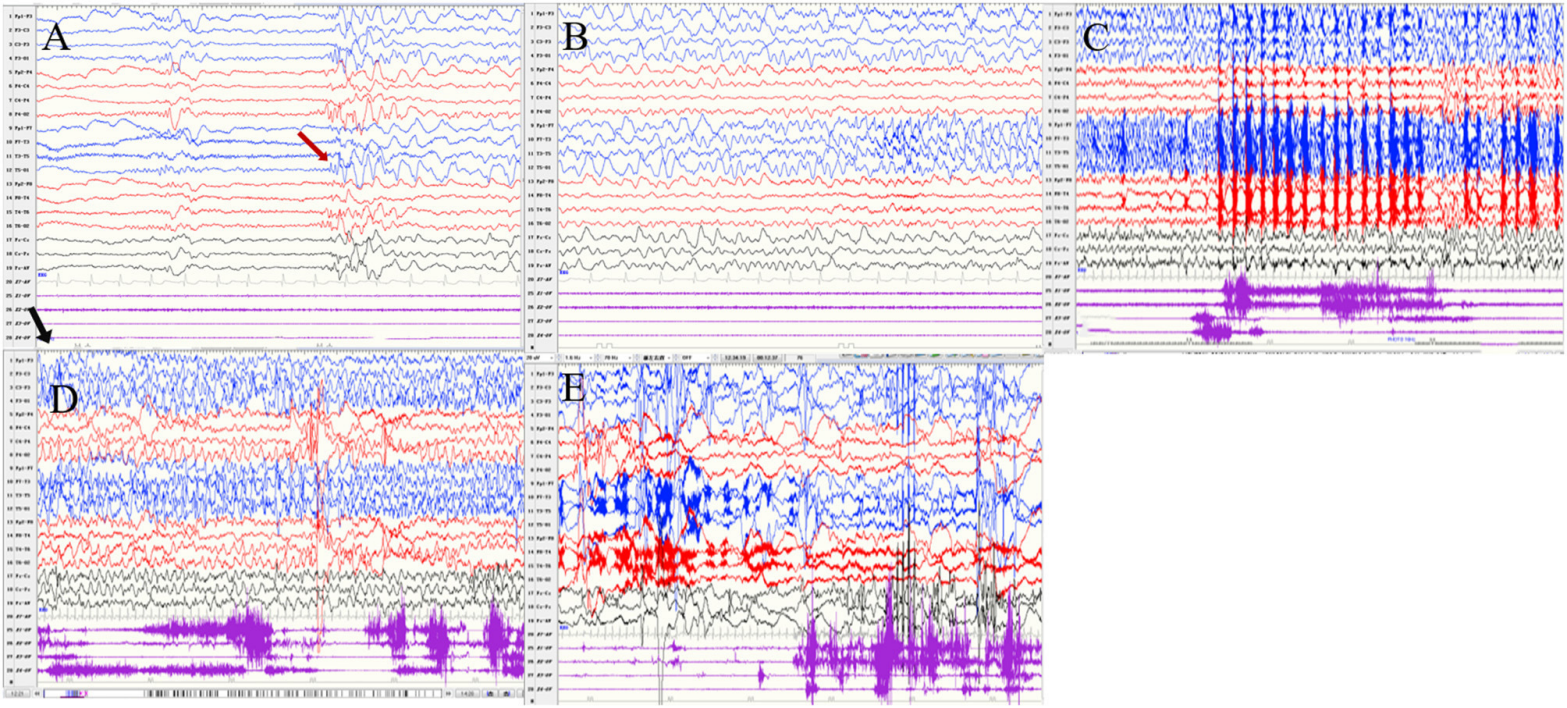
The ictal EEG of patient 4 **(A**–**E)** slow waves in the left temporal region → sharp wave rhythm in the left temporal region spreading to the contralateral temporal and frontal regions → irregular spikes mixed slow waves in the left hemisphere (The red arrow represents the origin of the seizure; the black arrow represents the starting point of the IPS.) (EEG, electrocardiogram; IPS, intermittent photic stimulation; X1, left deltoid; X2, right deltoid; X3, left quadriceps; X4, right quadriceps).

Seizures types of PCR included focal seizures (patients 1, 3, and 4), FBTCS (patient 2), and myoclonic seizures (patient 3). Focal seizures or FBTCS were evoked by eyes opened IPS in all patients, and the susceptive frequency was mainly concentrated in the range of 10–16 Hz. The origin of focal seizures or FBTCS were located on the left side, with left anterior and mesial temporal area in patient 1, posterior temporal area in patient 2 (Figure 3), and left temporal area in patient 3 (Figure 4) and patient 4 (Figure 5), respectively. The seizures lasted from 1 to 4 min. The ictal EEG of these four patients could be summarized into two forms. Of these, the ictal EEG of two patients was the onset of slow waves in the temporal area without spreading to generalization (patients 1 and 4), and the other one was fast rhythmic activities in the temporal area, spreading to the occipital area or gradually evolving into the generalization (patients 2 and 3). The brain MRI was normal in all patients.

### Antiseizure Medications and Follow-Up

All patients were treated with antiseizure medications. At the last follow-up, patient 1 used three antiseizure medications: valproic acid, lamotrigine, and carbamazepine and had no seizures for 4 years. Patient 2 was given lamotrigine and had no seizures for more than 5 years. The follow-up EEG revealed grade III PPR and IPS did not elicit seizure. Patient 3 was given valproic acid and had no seizures for 1 year. Patient 4 was given three antiseizure medications, valproic acid, levetiracetam, and oxcarbazepine, with occasional seizures. The psychomotor development of all patients was normal.

## Discussion

Photosensitivity is usually observed in genetic (idiopathic) generalized epilepsy, with PPR characterized by generalized epileptiform discharges (8). However, a growing number cases of focal seizures induced by IPS have been reported, almost originating from the occipital lobe (9, 10). Furthermore, cases of photosensitive focal seizures starting at the temporal lobe have also been reported (4, 11). Until now, eight cases of temporal onset photosensitivity focal seizures have been reported (4–6, 11–14). We collected four patients in our study, which is the largest cohort reported to date.

The clinical manifestations of temporal onset photosensitivity focal seizures were similar to those of temporal lobe epilepsy, including aura, automatism, disturbance of consciousness, and secondary tonic-clonic seizures. Among the eight previously reported cases, four adults between the ages of 19 and 37 had aura symptoms, including three emotional auras and visceral-sensory aura (Table 3) (4, 11, 13, 14). In our patients, only patient 1 suffered from hallucination aura, which might be related to the child's inability to accurately express subjective feelings. Of the eight previously reported cases, three had oropharyngeal automatisms and four had FBTCS (4–6, 11–14). In our study, patient 4 developed automatic symptoms such as swallowing and smacking, while patient 2 had FBTCS. Patient 3 also had myoclonic seizures caused by IPS, a phenomenon reported by Fiore et al. (11).

**Table 3:** Summary of EEG findings of previously reported cases with temporal onset focal seizure induced by IPS.

**No**.	**EEG background**	**Interictal EDs**	**PPR grade**	**Frequency distribution of PPR (Hz)**	**Seizure type of PCR**	**Electrographic onset**	**Frequency distribution of PCR (Hz)**	**Ictal symptom**
Benbadis et.al. (4)	–	Mesiobasal anterior temporal	–	–	FS, FBTCS	Bilateral temporal	–	Emotional aura, loss of awareness, manual and orobuccal automatisms
Seddigh et al. (14)	–	Generalized EDs, focal anterior temporal EDs	–	–	FBTCS	Right temporal	–	Behavioral arrest followed by oroalimentary automatisms occasionally followed by focal to bilateral tonic-clonic seizure
Seddigh et al. (14)		Right posterior temporal	–	–	FS, FBTCS, eyelid myoclonus	Right posterior temporal	–	Emotional aura, ictal speech with occasional focal to bilateral tonic-clonic seizure
Isnard et al. (13)	–	–	–	–	FS	Right temporal	–	Emotional aura, ictal speech, dystonic posturing of left limbs
Thomas et al. (12)	–	–	I (right temporal) I (right temporal)	5–10 16–25	FS	Right anteriortemporal	11 15	Coughing, tachycardia, and nausea followed by repeated vomiting, altered consciousness
Fiore et al. (11)	–	–	– III	14–20 14–18	FS, myoclonus seizures	Right temporal	–	Visceral-sensory aura, loss of awareness, manual and orobuccal automatisms, dystonic posturing of left hand
Lee et al. (6)	Normal	Normal	–	–	FBTCS	Left posterior temporal	15	Decreased consciousness, forced turning of the head to the right and focal to bilateral tonic-clonic seizure
Bratt et al. (5)	Normal	Occasionally focal slow waves of right hemisphere	II	13–20	FS	Right centrotemporal	–	Decreased conscious

*EEG, electroencephalographic; IPS, intermittent photic stimulation; EDs, epileptiform discharges; PPR, photoparoxysmal response; PCR, photoconvulsion response; FS, focal seizures; FBTCS, focal to bilateral tonic-clonic seizures; I, temporoparietooccipital epileptiform discharges; II, temporoparietooccipital and spreading to frontal regions; III, generalized epileptiform discharges*.

In neuroimaging studies, five reported cases of temporal onset photosensitivity focal seizures were associated with structural abnormality or metabolic abnormalities. Among the five cases, one case had schizencephaly and pachygyria on brain MRI, one had temporal lobe sclerosis, one had right temporal lobe malformwereation, and two cases had normal brain MRI, but positron emission tomography-computed tomography (PET-CT) showed temporal lobe glucose hypometabolism (4–6, 11–14). In our patients, patient 1 had a history of recurrent febrile seizures. Combined with the clinical manifestations of epileptic seizures and result of EEG, the patient could be diagnosed as temporal lobe epilepsy. Although her MRI was normal, it cannot be ruled out that there is a structural change or metabolic abnormalities in the temporal lobe, and PET-CT is required if necessary.

In the previously reported cases, the origin of PCR was mainly located in the right temporal lobe. Interestingly, however, in our patients, the origin of PCR was the left temporal lobe (Table 3). Due to the limited number of reported cases, it is not possible to determine whether the majority of patients with focal photosensitivity epilepsy originating in the temporal area originate on the left or right side. Meanwhile, we summarized the ictal EEG of the four cases and found that one was the onset of slow waves in the temporal area and the other was fast rhythmic activity in the temporal area, spreading to the occipital area or gradually evolving into the generalized discharges, which had not been previously associated. The ictal EEG of temporal medial epilepsy can generally be described as focal rhythmic activity in the theta range (5–9 Hz) normally located in the anterior temporal region, while the ictal EEG of lateral temporal epilepsy can commonly be described as polymorphic at 2–5 Hz in posterior temporal regions (15). According to description of the onset waves, patients 1 and 4 seemed to have a lateral temporal lobe epilepsy, patients 2 and 3 seemed to have a medial temporal epilepsy. Paradoxically, focal seizure of patient 1 originated in the anterior and mesial temporal area, and seizure of patient 2 originated in the posterior temporal area. This might be due to the close anatomical location of the medial and lateral temporal lobe, which made it difficult to accurately distinguish the origin of the two main subtypes in the scalp EEG. The frequency range of PCR was 10–16 Hz, which is consistent with previously reported cases (5, 6).

Inoue et al. proposed two mechanisms causing photosensitive temporal lobe seizures: (1) photodriving occipital pathways and (2) afferent pathways from the extraocular muscles or orbicularis oculi (16). In 1996, Benbadis et al. reported the first confirmed case of temporal lobe epilepsy completing subdural electrode recordings, in which photosensitive seizures were shown to originate from the right hyperexcitable temporal cortex, without early ictal involvement of the occipital visual cortex (4). However, a researcher using stereo-EEG recordings pointed out that photosensitive temporal focal seizures could be associated with the occipital cortex below the calcarine sulcus and could spread to mesiotemporal structures (13). This might be confirmed in the scalp EEG of our patients 2 and 3, as the ictal EEG of our patients 2 and 3 showed that the temporal discharges spread to the occipital area. However, the mechanism of photosensitive focal seizures started from the temporal lobe is still unclear, and more research is needed to study its pathogenesis in the future.

More and more scholars believed that there was a coexistence of focal discharges of the occipital origin and generalized discharges in photosensitive seizures (17–19). In a series of patients with genetic (idiopathic) generalized epilepsy, focal interictal discharges and focal seizures had been observed in 35% of patient population (20). In our previous studies of idiopathic epilepsy with photosensitive seizures, more than 70% of patients' PCR had FBTCS derived from the occipital lobe, and 50% of patients' PCR had myoclonic seizures with comprehensive network activation (9). In our study, PPR of all patients mainly showed generalized discharges. PCR of all patients showed focal seizures/FBTCS originating from the temporal area, and PCR of patient 3 also showed myoclonic seizures, which provided evidence for comprehensive network activation. All the above suggested that the focal discharges originating from the temporal area or occipital area could coexist with generalized discharges in photosensitive seizures. Therefore, there are increasing reasons to believe that photosensitive epilepsy should be classified as “systemic epilepsy” rather than generalized or focal epilepsy (9, 21). However, due to the limited number of patients, we could not derive the common clinical features, effective antiseizure medications, and prognosis of photosensitive temporal lobe seizure.

## Conclusion

Focal photosensitive seizures of temporal origin are very rare in photosensitive epilepsy. To our knowledge, this is the largest number of cases showing detailed evidence of temporal onset focal seizures induced by IPS compared with previous literatures. The ictal EEG of these four patients could be summarized into two forms, one was the onset of slow waves in the temporal area, and the other one was fast rhythmic activities in the temporal area, spreading to the occipital area or gradually evolving into the generalized discharges. In photosensitive seizures, both the focal discharges originating from the temporal area and the occipital area can coexist with generalized discharges, suggesting that photosensitive seizures are a continuum between focal and generalized seizures.

## Data Availability Statement

The original contributions generated for the study are included in the article/supplementary material, further inquiries can be directed to the corresponding author/s.

## Ethics Statement

The studies involving human participants were reviewed and approved by Biomedical Research Ethical Committee of Peking University First Hospital. Written informed consent to participate in this study was provided by the participants' legal guardian/next of kin. Written informed consent was obtained from the minor(s)' legal guardian/next of kin for the publication of any potentially identifiable images or data included in this article.

## Author Contributions

ZY conceptualized and designed the study, coordinated the study overall, and revised the manuscript. YN co-designed the study, drafted the initial manuscript, and revised the manuscript. PG, XJ, and HY helped to collect and summarize data and revised the manuscript. All authors approved the final revision of the article.

## Conflict of Interest

The authors declare that the research was conducted in the absence of any commercial or financial relationships that could be construed as a potential conflict of interest.

## Publisher's Note

All claims expressed in this article are solely those of the authors and do not necessarily represent those of their affiliated organizations, or those of the publisher, the editors and the reviewers. Any product that may be evaluated in this article, or claim that may be made by its manufacturer, is not guaranteed or endorsed by the publisher.
